# Unveiling the structural mechanisms behind high affinity and selectivity in phosphorylated epitope-specific rabbit antibodies

**DOI:** 10.1016/j.jbc.2024.107989

**Published:** 2024-11-13

**Authors:** Keisuke Kasahara, Raiji Kawade, Makoto Nakakido, Ryo Matsunaga, Hiroki Akiba, Kevin C. Entzminger, Toshiaki Maruyama, Shigeru C.J. Okumura, Jose M.M. Caaveiro, Daisuke Kuroda, Kouhei Tsumoto

**Affiliations:** 1 Department of Bioengineering, School of Engineering, The University of Tokyo, Bunkyo-ku, Tokyo, Japan; 2 Department of Chemistry and Biotechnology, School of Engineering, The University of Tokyo, Bunkyo-ku, Tokyo, Japan; 3 Center for Drug Design Research, National Institutes of Biomedical Innovation, Health and Nutrition, Ibaraki City, Osaka, Japan; 4 Graduate School of Pharmaceutical Sciences, Kyoto University, Sakyo-ku, Kyoto, Japan; 5 Abwiz Bio Inc., San Diego, California, USA; 6 Laboratory of Protein Drug Discovery, Graduate School of Pharmaceutical Sciences, Kyushu University, Fukuoka-shi, Japan; 7 Research Center for Drug and Vaccine Development, National Institute of Infectious Diseases, Shinjuku-ku, Tokyo, Japan; 8 Medical Proteomics Laboratory, Institute of Medical Sciences, The University of Tokyo, Minato-ku, Tokyo, Japan

**Keywords:** antibody, antigen, posttranslational modification, protein structure, peptide interaction, molecular dynamics

## Abstract

Protein phosphorylation is a crucial process in various cellular functions, and its irregularities have been implicated in several diseases, including cancer. Antibodies are commonly employed to detect protein phosphorylation in research. However, unlike the extensive studies on recognition mechanisms of the phosphate group by proteins such as kinases and phosphatases, only a few studies have explored antibody mechanisms. In this study, we produced and characterized two rabbit monoclonal antibodies that recognize a monophosphorylated Akt peptide. Through crystallography, thermodynamic mutational analyses, and molecular dynamics simulations, we investigated the unique recognition mechanism that enables higher binding affinity and selectivity of the antibodies compared to other generic proteins with lower binding affinity to phosphorylated epitopes. Our results demonstrate that molecular dynamics simulations provide novel insights into the dynamic aspects of molecular recognition of posttranslational modifications by proteins beyond static crystal structures, highlighting how specific atomic level interactions drive the exceptional affinity and selectivity of antibodies.

Specific residues of proteins are frequently subject to posttranslational modifications through interactions with specific enzymes. These modifications can impact various properties of proteins, including their structure, dynamics, and activity in biological processes (1). Phosphorylation, among many types of modifications, plays a significant role in a wide range of cellular processes (2, 3). Even a single phosphorylation can switch protein activities on or off in signaling pathways, and abnormal phosphorylation is linked to various diseases, such as cancer (4, 5).

Antibodies are widely used as a high-sensitivity platform to detect phosphorylation of specific proteins in various research fields. These antibodies are typically obtained by immunizing host animals with a phosphorylated epitope or by using synthetic antibody libraries. One of the most effective methods to understand the atomic interactions between antibodies and antigens is to determine the crystal structures of their complexes. However, while the number of crystal structures of antibodies has been rapidly increasing (6), the number of antibody-phosphorylated epitope complexes is still limited, resulting in a poor understanding of the interactions between antibodies and phosphorylated epitopes.

Phosphorylated amino acids have an important characteristic of equilibration. Although previous studies have often assumed that phosphorylated residues are in the unprotonated state (PO_3_^2−^) (7), the phosphate group (with a pKa of ∼6) exists in an equilibrium mixture of non-protonated (PO_3_^2−^) and singly protonated (PO_3_H^−^) states at physiological pH (8, 9). Our previous computational study on non-antibody proteins showed that even a single protonation could affect the dynamics and recognition of a phosphorylated serine residue (10). Thus, to gain molecular insights into the recognition mechanism of phosphorylated amino acids by antibodies, it is necessary to consider both protonation states.

In this study, we generated four rabbit monoclonal antibodies by immunizing a rabbit and obtained crystal structures for two of them. These two antibodies showed different characteristics against their antigen, a phosphorylated Akt peptide. One exhibited the highest binding affinities among the four antibodies generated to both phosphorylated and non-phosphorylated peptides, while the other showed the highest selectivity, recognizing the phosphorylated peptide but exhibiting no detectable binding to the non-phosphorylated peptide. Based on the crystal structures, we conducted mutational analyses using isothermal titration calorimetry (ITC) and molecular dynamics (MD) simulations in different protonation states to evaluate the contribution of each residue to recognition. Our results showed that the recognition mechanisms of the antibodies, which possessed nanomolar binding affinities, were significantly different from those of generic proteins that showed micromolar affinities. These findings demonstrate that MD simulations can provide new insights into the dynamic aspects of molecular recognition of posttranslational modifications by proteins, which cannot be obtained through static crystal structures alone, and illustrate how specific interactions at the atomic level contribute to the remarkable binding affinity and selectivity of antibodies.

## Results

### Generation of rabbit single-chain variable fragment specific to a phosphorylated serine residue

We generated four clones with high selectivity against the phosphorylation on 473-Ser of the Akt peptide (RPHFPQF[pS]YSAS) through rabbit immunization followed by phage display and the subsequent ELISA assays (Table S1). In order to investigate the molecular details of the antigen-binding sites, the IgG molecules were converted to the single-chain variable fragment (scFv) format.

The kappa light chain of rabbit antibodies has a unique characteristic: an interdomain disulfide bond between the variable domain (80-Cys of the light chain by Chochia definition (11)) and constant domains (170-Cys of the light chain). However, when the sequences of variable domains (V_H_, V_L_) of a rabbit antibody are extracted as an scFv format, the 80-Cys loses its partner in the disulfide bond, and the free 80-Cys can destabilize the scFv. While a previous study has shown that the disulfide bond does not affect the binding affinity to an antigen, it contributes to the thermal stability of a rabbit antibody (12). To address this issue and obtain a stable scFv construct, we mutated the 80-Cys of the light chain to a Ser residue, resulting in significant stabilization of the scFv format (Fig. S1).

### Binding affinity and selectivity of the rabbit scFvs for the phosphorylated peptide

**Figure 1:**
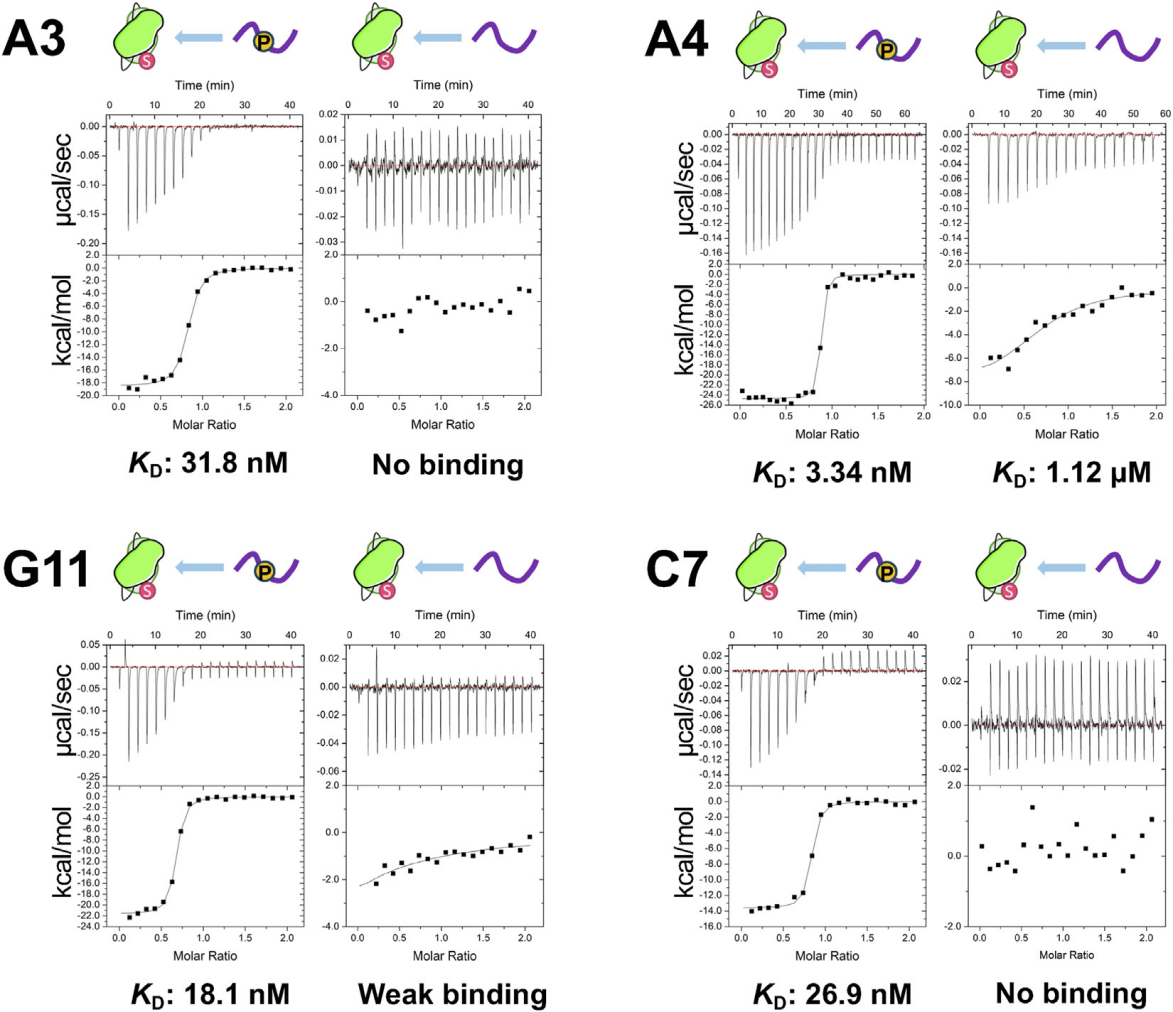
**Binding activities and selectivity of rabbit antibodies used in this study**. The peptide antigens were prepared with or without a phosphorylated serine residue.

To quantify the binding affinity and selectivity of each clone toward phosphorylated and non-phosphorylated peptide antigens, we utilized ITC (Fig. 1 and Table S2). From the thermodynamic parameters obtained, two clones demonstrated higher activity and selectivity. Specifically, the antibody A4 exhibited the highest binding affinity of 3.34 nM toward the phosphorylated antigen and 1.12 μM affinity toward the non-phosphorylated antigen, which corresponds to a selectivity difference of approximately 300 times between the phosphorylated and non-phosphorylated peptides (Fig. 1). Another clone, C7, displayed the highest selectivity toward the phosphorylated antigen with a binding affinity of 26.9 nM, whereas no measurable binding to the non-phosphorylated antigen was observed (Fig. 1).

### X-ray crystallography of the rabbit scFv in complex with the phosphorylated antigen

**Figure 2:**
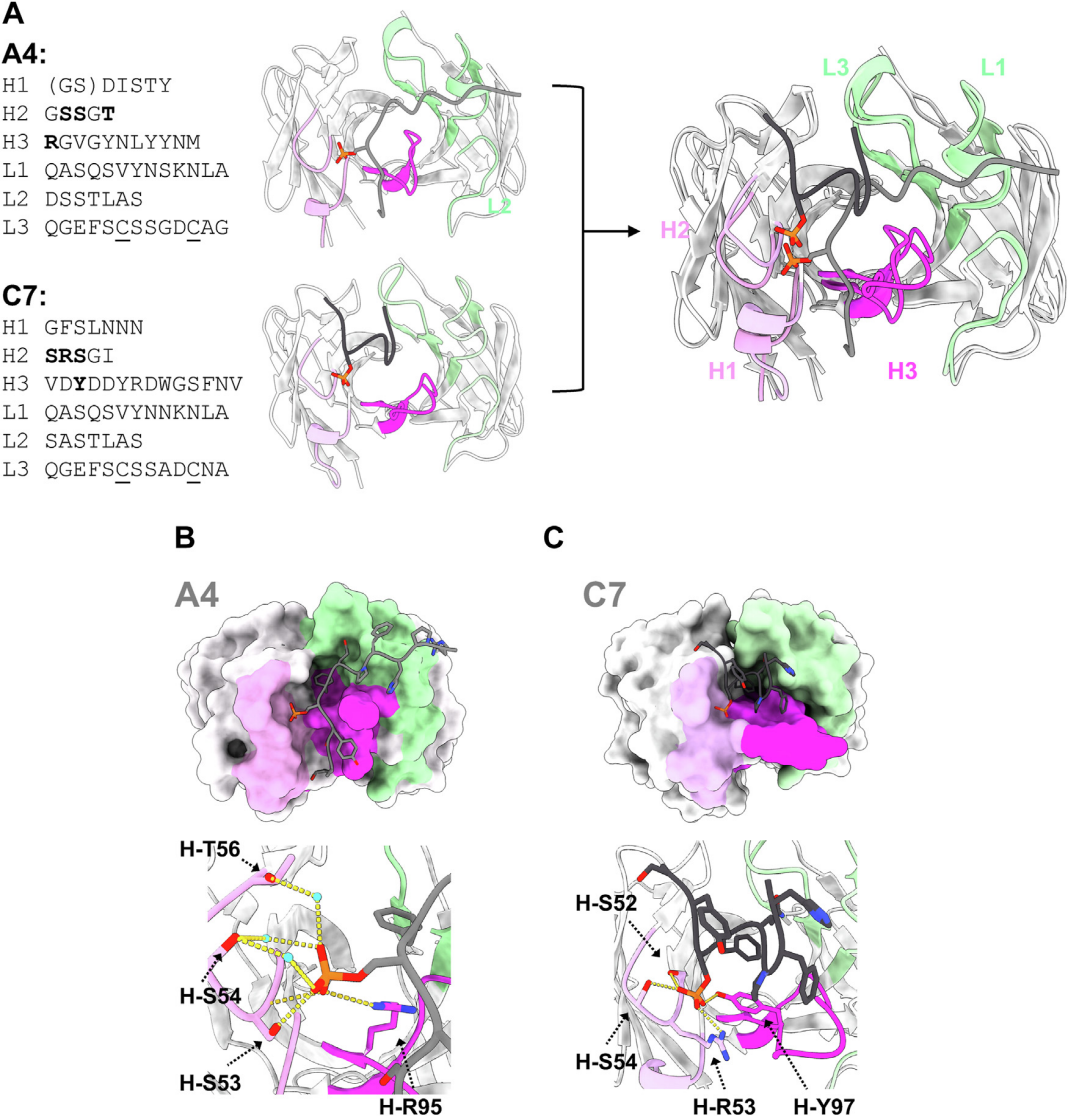
**Crystal structure of rabbit antibodies (A4 and C7) in complexed with the phosphorylated peptide.***A*, superposition of the crystal structures of the antibodies (A4 scFv and C7 Fab) and their CDR sequences. In the sequence panels, disulfide bonding cysteine residues are underlined, missing residues in the crystal structures are shown in the *parenthesis*, and the interacting residues with the phosphate group are *bolded*. The peptide is colored *gray* and *black*, respectively. *B, C,* schematic illustration of the antigen–antibody interactions of A4 scFv (*B*) and C7 Fab (*C*). The antibodies are shown as surface representations. Water molecules are colored in *cyan*. Potential hydrogen bond interaction (<3.5 Å distance between hydrogen bond donor and acceptor) is shown in a *yellow dotted line*. For antibody numbering, the Chothia numbering scheme was adapted throughout the manuscript.

To better understand the underlying binding mechanisms that confer higher affinity and selectivity, we determined the crystal structures of two scFv (A4 and C7) and a Fab (C7) in complex with the phosphorylated antigen (Fig. 2 and Table S3). We crystallized the complex with the Fab of C7 to corroborate the structure of the complex of the scFv construct, since the latter displayed poor resolution (2.8 Å) and non-crystallographic symmetry resulting in poor R-factors (see below).

The overall Fv structures of A4 and C7, except complementarity determining region (CDR)-H3, are quite similar to each other (Fig. 2*A*, with Cα-root mean square deviation (RMSD) values of 0.74 Å and 0.72 Å for the aligned residues of the heavy and light chains, respectively). Both antibodies possess a 13-residue CDR-L3, in which a disulfide bond is formed between L-94 and L-95D (Chothia numbering). This is a common characteristic of rabbit antibodies.

In the A4 antibody–antigen complex, all CDRs except for CDR-L2 contributed to the peptide recognition to varying extents, although two residues at the N-terminal region of CDR-H1 were not visible (Fig. 2*A*). Notably, the Arg residue at CDR-H3 (H-R95) formed a salt bridge or hydrogen bond with the oxygen atoms of the phosphate group, while the side chain and the backbone amide of the Ser residue at CDR-H2 (H-S53) formed hydrogen bonds with an oxygen atom of the side chain of the phosphorylated Ser residue. Despite the high selectivity, only these two residues, H-R95 and H-S53, are within a hydrogen bonding distance (3.5 Å between donor and acceptor) from the phosphate group (Fig. 2*B*). Even when considering more loosely defined physical contacts (less than 4.0 Å from the phosphate group) rather than strictly hydrogen bonds, only a few carbon atoms in CDR-H2 (H-S33, H-G52 and H-S53) are newly included as atoms that contact the phosphate group. It is worth noting that the crystal structure of A4 has a high enough resolution (1.4 Å) to observe many water molecules at the antibody–antigen interface, indicating additional interactions between the phosphorylated residue and two residues of CDR-H2 (H-S54 and H-T56) through water-mediated interactions (Fig. 2*B*). Besides the phosphorylated Ser residue, a Phe residue in the middle of the peptide antigen buried its sidechain at the V_L_/V_H_ interface, and the adjacent Gln residue of the peptide antigen formed hydrogen bonds with the backbone of CDR-L3, contributing to the overall stability of the complex.

Compared to the A4 complex, the crystal structure of the C7 scFv–peptide complex is of lower quality (2.8 Å resolution), with only six out of the 12 peptide residues visible, including the phosphorylated Ser residue. This suggests that both N-terminal and C-terminal residues of the peptide are highly flexible even after antibody binding, and they contribute less to the antibody–antigen interaction. To address the lower quality of the C7 scFv crystal structure, we also determined the crystal structure of the Fab format. Although the quality of this structure improved very significantly (1.33 Å resolution), the overall conformations of both the peptide antigen and the C7 antibody, including the orientations of their side chains, were nearly indistinguishable (Cα-RMSD for the aligned residues of the heavy chain, light chain, and peptide were 0.52 Å, 0.54 Å, and 0.62 Å, respectively). These values are well within the differences found for crystals of the same protein crystallized under different buffer conditions. Interestingly, some of the N-terminal and C-terminal residues of the peptide antigen could not be modeled in the electron density of the crystal structure of the C7 Fab-phosphorylated peptide complex (Fig. 2*C*), confirming the intrinsic flexibility of these regions in the interaction with the C7 antibody. The C7 antibody has a 14-residue CDR-H3, in which six residues are within 4 Å distance from the peptide antigen, and the hydroxy group of a Tyr residue (H-Y97) is within hydrogen bonding distance (2.6 Å) of the phosphate group of the antigen (Fig. 2*C*). Additionally, the Arg residue at CDR-H2 (H-R53) interacts with the phosphate group of the antigen. Furthermore, two adjacent Ser residues (H-S52 and H-S54) at CDR-H2 form hydrogen bonds with the phosphate group, with all residues of CDR-H2, except Gly, positioned within a 4 Å distance from the antigen. Similar to the A4 antibody, apart from the phosphorylated Ser residue, a Phe residue in the peptide has a buried sidechain at the V_L_/V_H_ interface, with a hydrogen bond between a His residue at CDR-H2 (H-H58) and the backbone of the Phe residue in the peptide (Fig. 2*C*). CDR-L3 also interacts with the peptide antigen, with a single hydrogen bond between the backbone of Glu residue (L-E91) at CDR-L3 and the sidechain of the Gln residue in the peptide.

In summary, the static crystal structures demonstrated that both antibodies, A4 and C7, possess a basic residue (Arg) and a few uncharged polar residues (Ser and Thr) to recognize the phosphate group of the peptide antigens, which is reminiscent of non-antibody proteins that recognize a phosphorylated amino acid (10). Interestingly, despite differences in the overall conformation of the peptide when binding to A4 or C7, the phosphorylated Ser, along with Gln and Phe residues, occupy similar positions spatially at the antibody–antigen interfaces (Fig. 2*A*). This results in similar peptide interactions with the antibodies.

### Mutational analysis of how charged and uncharged polar residues contribute to binding of the phosphate group

Crystal structures of the antibodies revealed common types of amino acid residues involved in recognizing the phosphate group, specifically small uncharged polar residues (Ser/Thr) and a charged basic residue (Arg). To assess the contributions of individual residues to the recognition of phosphorylated serine, we generated several alanine mutants of the heavy chain (S53 A, S54 A, T56 A, and R95 A for the antibody A4, and S52 A, R53 A, and S54 A for the antibody C7) and analyzed their binding using ITC (Table 1).

**Table 1:** Thermodynamic profiles of wildtype and mutant antibodies interacting with the phosphopeptides

Antibody	*K*_D_ (nM)	Δ*G* (kcal/mol)	Δ*H* (kcal/mol)	*T*Δ*S* (kcal/mol)
A4 WT	3.34 ± 0.63	−11.6 ± 0.1	−24.4 ± 0.4	−12.8 ± 0.4
A4 H-S53A	3.55 ± 1.72	−11.6 ± 0.3	−24.9 ± 0.5	−13.3 ± 0.3
A4 H-S54A	6.95 ± 2.32	−11.2 ± 0.2	−23.5 ± 0.2	−12.3 ± 0.4
A4 H-T56A	4.00 ± 0.89	−11.5 ± 0.1	−23.0 ± 0.6	−11.5 ± 0.6
A4 H-R95A	No binding			
C7 WT	26.9 ± 14.2	−10.4 ± 0.4	−14.4 ± 0.7	−4.0 ± 1.0
C7 H-S52A	664 ± 34	−8.4 ± 0.0	−11.1 ± 0.1	−2.7 ± 0.1
C7 H-R53A	No binding			
C7 H-S54A	95.3 ± 20.9	−9.6 ± 0.1	−13.3 ± 0.5	−3.7 ± 0.6

Previous studies have suggested that positively charged residues and small uncharged polar residues are important in recognizing phosphorylated amino acids (10). In antibody A4, charged residue H-R95 and small uncharged polar residues, such as H-S53, S54, and T56, are located near the phosphate group of the peptide antigen (Fig. 2*B*). The H-R95 A mutant showed a drastic loss of binding activity, indicating that H-R95 is a hot spot for binding. H-S53 makes direct hydrogen bonds with the phosphate group, but there was no significant difference in binding activity between the wildtype (WT) and H-S53 A mutant. Similarly, the H-S54 A and H-T56 A mutants showed binding affinities toward the antigen that were within the experimental errors of the WT (Table 1).

Antibody C7 also has a charged residue, H-R53, and small polar residues, such as H-S52 and H-S54, near the phosphate group. As expected, the loss of the basic residue (H-R53 A) caused a critical loss of binding activity. However, in contrast to antibody A4, we observed significant decreases in binding activity for C7 with mutations of uncharged polar residues. As compared to the WT, the binding affinity was 25 and 4 times smaller for the H-S52 A and H-S54 A mutants, respectively (Table 1). These results suggest that while charged residues determine binding activity, uncharged polar residues also play a role in the selectivity of antigen recognition.

### MD analysis of how the antibodies A4 and C7 recognize the phosphate groups

Mutagenesis combined with ITC measurements suggested that the functional differences between the antibodies A4 and C7 (*i.e.*, the former binds to both phosphorylated and non-phosphorylated antigens, while the latter binds only to the phosphorylated antigen) may be attributed to the roles of small uncharged polar residues. However, the specific roles of these interactions remain unclear when considering only the static crystal structures. To investigate the dynamics of the antibody–antigen interactions, we conducted triplicate 1 μs MD simulations of the A4 and C7 scFv antibodies in complex with the phosphorylated antigen, respectively. Before the simulations, missing residues in the crystal structures were complemented through CHARMM-GUI (13). To see the effect of the protonation state of the phosphate group, we also employed two protonation states of the phosphate group (PO_3_H^−^ and PO_3_^2−^).

To confirm the convergence of the simulation, we first computed the RMSD of the Cα atoms of the antibodies, except for the GS linker and first and last five residues at the N-/C-terminal residues, with respect to the first snapshot of each trajectory (Fig. S2). The Cα-RMSD values of A4 and C7 were quite stable during each simulation. We discarded the initial 200 ns trajectories and used the remaining 800 ns trajectories for the subsequent analysis.

To evaluate how each residue of the antibodies interacts with the phosphate group during the simulation, we examined the frequencies of these interactions. Following the criteria from a previous study (10), we defined a protein residue as interacting with phosphoserine if the distance between the phosphorus atom and any atom of the residue was less than 4.0 Å. The results are summarized in Table 2. Interestingly, in the simulations of antibody A4, eight residues interact with the phosphoserine at frequencies greater than 10%, whereas in the C7 antibody, only four such residues emerged. It is also worth noting that in antibody A4, the frequency of interactions varied from 15% (H-T56) to more than 98% (H-S53). In contrast, the four interactions identified in the simulations of antibody C7 are almost 100% conserved.

**Table 2:** Interactions between the phosphorus atom and nearby residues in the A4 and C7 antibodies

Residue	Number	PO_3_H^−^ (%)	PO_3_^2−^ (%)
A4			
TYR	H32	0.1	0.0
SER	H33	60.6	61.0
HIS	H50	34.7	33.9
GLY	H52	83.2	96.2
SER	H53	87.6	98.4
SER	H54	48.7	53.1
GLY	H55	20.0	28.0
THR	H56	15.0	0.2
TYR	H58	4.1	0.0
ARG	H95	63.3	80.6
TYR	H99	0.5	0.0
C7			
SER	H52	99.9	100.0
ARG	H53	100.0	100.0
SER	H54	100.0	100.0
ILE	H56	0.3	0.8
TYR	H97	100.0	100.0

Interactions are counted if distances between the phosphorus atom and any atoms in surrounding residues are less than 4.0 Å. Frequencies were averaged over three independent simulations for each system.

More specifically, in the crystal structure of antibody A4, only two residues, H-S53 and H-R95, directly formed hydrogen bonds with the phosphate group. However, simulations revealed additional interactions with H-S33, H-H50, H-G52, H-S54, H-G55, and H-T56, identifying a total of eight residues interacting with the phosphoserine—six of which were absent in the static crystal structure. The MD simulations suggest that antibody A4 recognizes the phosphate group through a combination of a basic side chain (Arg), multiple uncharged polar residues (Ser and Thr), and main chains of residues with less bulky side chains, such as Gly.

In the crystal structure of antibody C7, the phosphate group formed hydrogen bonds directly with four residues: H-S52, H-R53, H-S54, and H-Y97. All of these were also observed in simulations to interact with the phosphate atom for almost 100% of the simulation time (Table 2).

**Figure 3:**
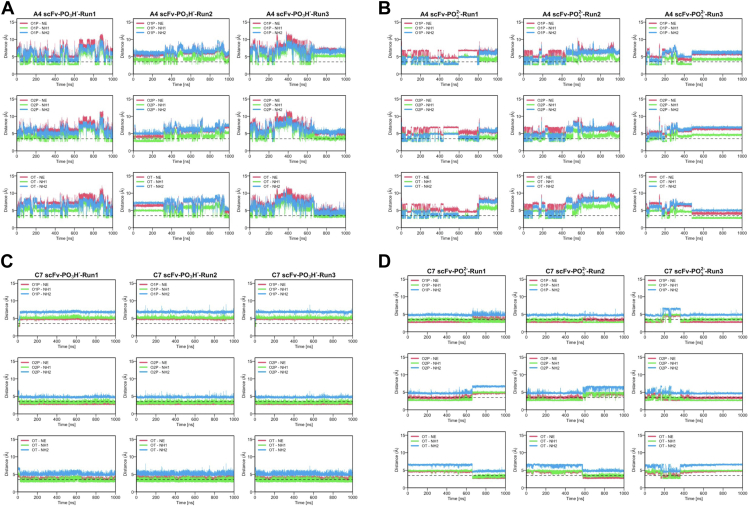
**Time evolution of bidentate interactions between an Arg residue (H-R95 for A4 and H-R53 for C7, respectively) and the phosphate group.***A*, A4 – PO_3_H^−^. *B*, A4 – PO_3_^2−^. *C*, C7 – PO_3_H^−^. *D*, C7 – PO_3_^2−^.

In a previous study, we demonstrated that a protein recognizing a phosphorylated Ser residue can form a bidentate interaction with an Arg residue in the binding site (10). To further investigate this, we analyzed how frequently such strong interactions occur in antibody–antigen complexes and whether they contribute to the selectivity of molecular recognition. Interestingly, although both antibodies exhibited these interactions (Table S4), antibody C7, which shows much higher selectivity, maintained the bidentate interaction for nearly 100% of the simulation time (Fig. 3 and Table S4). Taking a simulation of PO_3_^2−^ as an example, it was observed that the NE atom and the N1 atom at the terminal of H-R53 always formed hydrogen bonds with the two oxygen atoms of the phosphate group in the simulations of antibody C7. In contrast, antibody A4, which can bind to both phosphorylated and non-phosphorylated antigens with higher affinity than C7, exhibited the bidentate interaction less frequently (Fig. 3 and Table S4). The PO_3_^2−^ state showed a higher frequency of this interaction, similar to what was observed in a non-antibody protein (10).

These observations collectively indicate that the recognition mechanisms of the phosphorylated serine residue differ between antibodies A4 and C7. In A4 simulations, the interactions were variable, involving more residues than those observed in the crystal structure, whereas C7 consistently exhibited interactions between the limited residues in the paratopes and the phosphorylated residue (Table 2). These findings could reflect the binding assays from ITC, where antibody A4 was capable of recognizing both phosphorylated and non-phosphorylated epitopes, although with weaker affinity for the latter. In contrast, C7 could only recognize the phosphorylated epitope (Fig. 1 and Table S1).

### Comparative analysis of how the recognition of phospho-epitope by the antibodies A4 and C7 differ from non-antibody proteins

**Figure 4:**
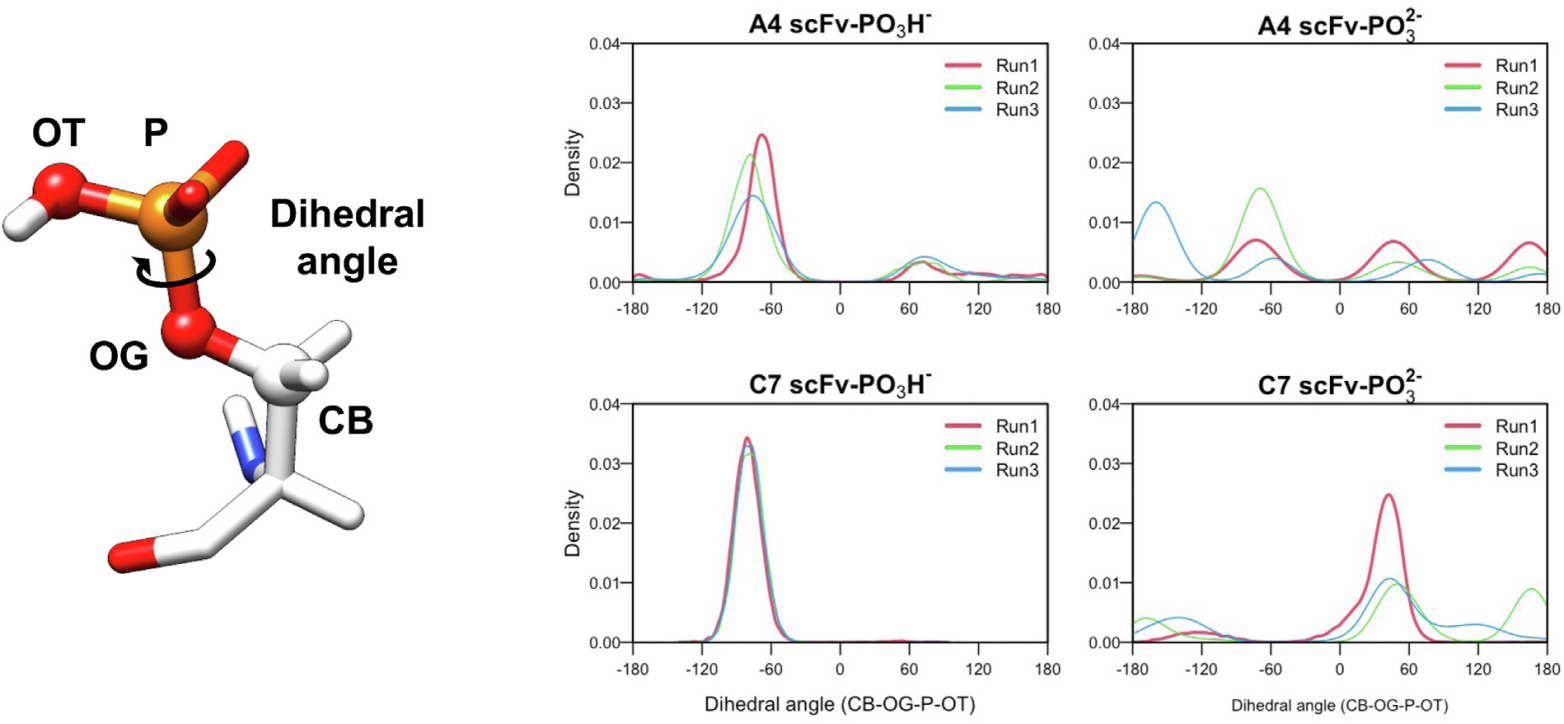
**Distribution of the dihedral angle of the phosphate group in singly protonated (PO**_**3**_**H**^**−**^**) and unprotonated (PO**_**3**_^**2−**^**) states during the MD simulations of the rabbit antibodies (A4 and C7).**

**Figure 5:**
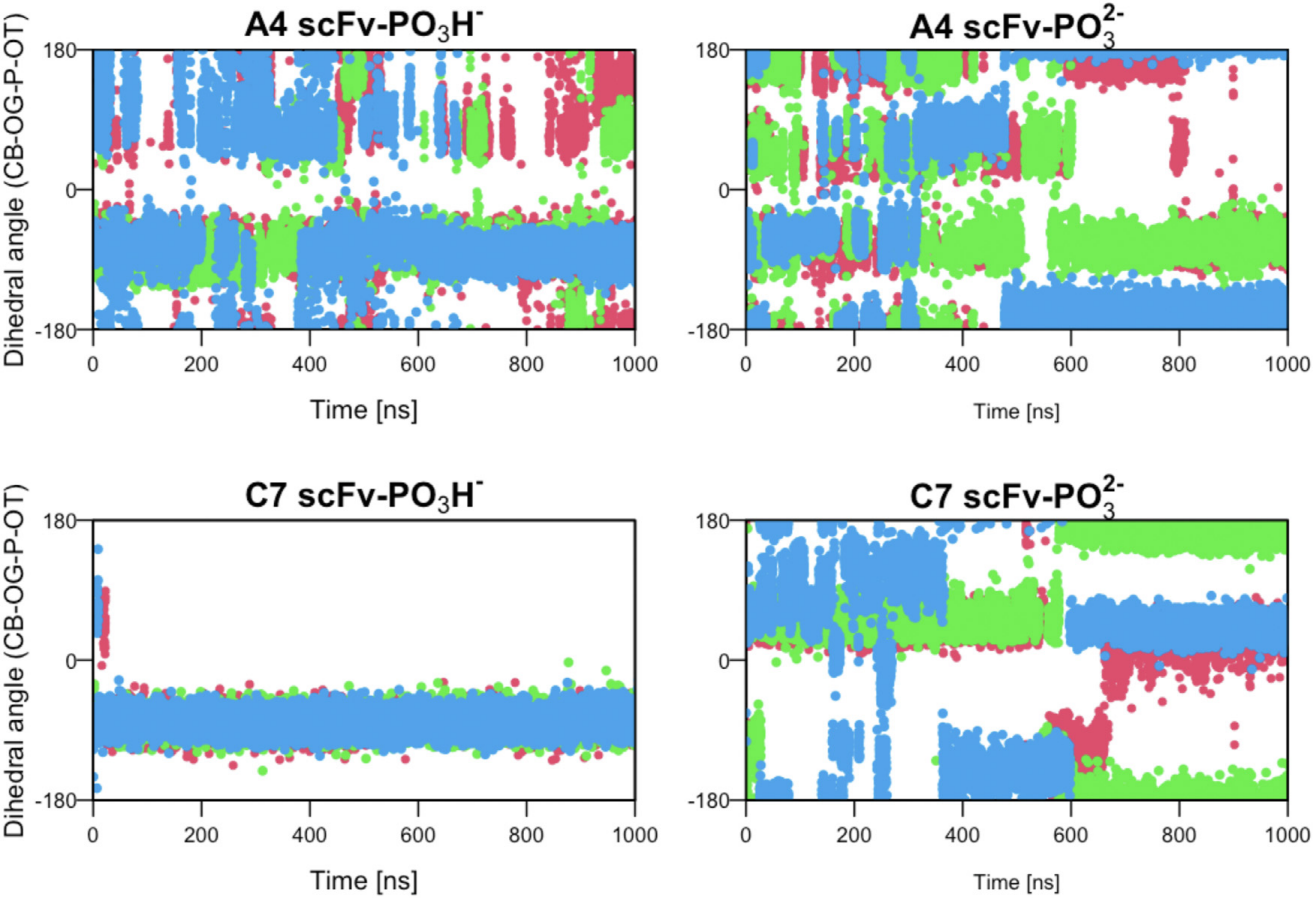
**Time evolution of the dihedral angle consisting of CB, OG, P, and OT atoms of the phosphate group during the simulations of the antibodies**. Different colors represent different replicates of the simulations.

In a previous study, it was suggested that non-antibody proteins recognize PO_3_H^−^ with a transition between two discrete conformational states, and they recognize PO_3_^2−^ through spontaneous rotation of the phosphate group of a phosphorylated serine residue, resulting in a favorable entropy change upon binding of the latter (10). The binding affinities of these proteins ranged from 0.24 to 28 μM, which are weaker than the antibodies studied in this study (3.34 nM and 26.9 nM for A4 and C7, respectively). We quantified this rotation of the phosphate group in the simulations by calculating the dihedral angle of the sidechain and used this to investigate the structural selectivity of the recognition between the antigen and antibodies A4 and C7 (Fig. 4 and 5). Interestingly, in the simulations with PO_3_H^−^, both A4 and C7 exhibited a single peak at the same angle (Fig. 4), indicating that the phosphate group was strictly fixed in a stable state by the antibodies. In particular, in C7, the phosphate group was almost never allowed to rotate during the simulations (Fig. 5). Overall, in the simulations with PO_3_H^−^, A4 and C7 fix the dihedral angle of the phosphate group of the antigen with a single peak. This suggests greater structural selectivity compared to non-antibody proteins, which exhibit weaker binding affinities and display two peaks in their simulations (10).

In contrast to PO_3_H^−^, the chemical structure of PO_3_^2−^ is symmetric with three equivalent oxygen atoms. Therefore, non-antibody proteins recognize a phosphorylated epitope with the phosphate group being constantly rotated by 120° (10). In the simulation of A4 with PO_3_^2−^, we observed three peaks in the distribution of the dihedral angle (Fig. 4). However, the peaks were not equivalent, indicating that the time scales of the rotations were varied. We confirmed the rotation by 120° in the simulations of antibody A4, which occurred in tens to hundreds of nanoseconds (Fig. 5), whereas in non-antibody proteins, it occurred on a much shorter time scale (Fig. S3). The antibodies demonstrated a tendency to decelerate the rotation of PO_3_^2−^, a phenomenon more pronounced with antibody C7. The distributions exhibited fewer peaks, suggesting that the phosphate group recognized by antibody C7 rotates less frequently (Fig. 4). Collectively, these results suggest that, regardless of the protonation state, antibodies A4 and C7 recognize the phosphate group in a more stereoselective manner compared to other generic proteins. This tendency is particularly pronounced in the case of antibody C7.

## Discussion

Several studies have discussed the mechanisms by which natural protein families, such as 14-3-3 and BRCT, capture and discriminate the phosphate group (14, 15). However, there is limited research on how antibodies accomplish this task, with only a few studies analyzing interactions through cocrystallization with phosphorylated peptides (16, 17, 18, 19, 20, 21, 22). To enhance our understanding of the molecular mechanisms underlying phospho-specific epitope recognition by antibodies, we conducted further analysis using a combination of experimental and computational techniques to evaluate the contribution of each paratope residue in the recognition of a phosphorylated epitope.

From the structural and dynamic analysis of antibodies A4 and C7, we have identified some common features and characteristics. First, both antibodies utilize a basic residue, Arg, to recognize the phosphate group. When we mutated Arg to Ala, the binding to the phospho-epitope was completely lost, indicating that Arg plays a crucial role in the recognition of the phospho-epitope. Second, both antibodies form several hydrogen bonds with uncharged polar amino acids and the main chain's nitrogen atom (Fig. 2). These amino acid composition characteristics of the paratopes are reminiscent of non-antibody proteins that recognize phosphorylated epitopes (10). Therefore, the interactions through a charged residue and several uncharged polar residues appear to be a common mechanism that nature has evolved to interact with phosphorylated proteins.

**Figure 6:**
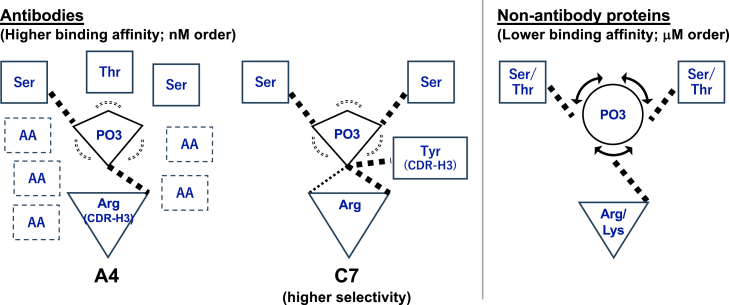
**Proposed model for molecular recognition of a phosphorylated Ser residue**. Residues in *solid rectangles* represent interacting residues observed in the crystal structures. 'AA' in *dotted rectangles* indicates amino acid residues interacting with the phosphate group that are not visible in static crystal structures. These interactions are specifically observed in the A4 antibody, which binds to both phosphorylated and non-phosphorylated antigens, thereby restricting the rotation of the phosphate group. In contrast, the C7 antibody, which binds only to phosphorylated antigens, more effectively restricts the rotation of the phosphate group through bidentate interactions with the Arg residue. These interactions are not present in the crystal structure but are observed during simulations. Non-antibody proteins, on the other hand, recognize the phosphate group solely through a charged residue and small hydrophilic residues, allowing for continuous rotation of the phosphate group.

In addition, in the simulations, the dihedral angle of PO_3_H^−^ was observed to be limited to a single peak (Fig. 4). This observation suggests that both antibodies exhibit much higher structural specificity than non-antibody proteins, which show transitions between two states of the phosphate group (10). Moreover, the dihedral angle of PO_3_^2−^ showed much less uniform distribution in the simulations of the antibodies compared to non-antibody proteins, suggesting that the antibodies tend to suppress the rotations of the phosphate group, even when it is in the symmetric PO_3_^2−^ state (Figure 4, Figure 5). Therefore, it is tempting to speculate that non-antibody proteins, which exhibit weaker binding affinities, perceive the phosphate group as a “charged globular residue”. This allows the phosphate to roll over the surrounding interacting residues due to the unidirectional nature of the interactions. In contrast, antibodies with higher binding affinities and selectivity perceive the phosphate group as a “charged branched residue”, requiring more stereospecific recognition (Fig. 6).

On the other hand, some differences are also observed between antibody A4 and C7. A4, which exhibits the highest binding affinities toward both phosphorylated and non-phosphorylated antigens, has many interacting residues that alternately bind to the phosphate group (Table 2). In stark contrast, C7, which demonstrates much higher selectivity (*i.e.*, it binds to the phosphorylated antigen but does not bind to the non-phosphorylated antigen) (Fig. 1 and Table S2), forms bidentate hydrogen bonds with the phosphate group *via* an Arg residue (Fig. 3 and Table S4). This bidentate bond is thought to be one of the strongest interactions between residues (23) and is a reason that the rotation of the phosphate group is particularly suppressed by antibody C7, which uses only four residues that always form six or more hydrogen bonds with the phosphate group. Through these stable noncovalent bonds, C7 prevents the phosphate group from rotating, resulting in a high strict selectivity of the phosphorylated epitope over the non-phosphorylated one.

From these observations, we may propose a design principle for phospho-specific antibodies: incorporating a single Arg residue with a molecular surface composed of uncharged polar residues around the Arg may make the protein specific to the phosphorylated epitope. This could result in a binding affinity in the order of nM with several thousand times higher selectivity over non-phosphorylated epitopes. In a previous study, Koerber *et al.* were able to design antibodies that recognize phosphorylated peptides (24). They achieved this by incorporating an existing anion-binding motif, consisting of three consecutive residues where multiple main-chain amides form hydrogen bonds with the anion, into CDR-H2 of antibodies that do not naturally recognize phosphorylated peptides. While their nature-inspired strategy might have broader applications due to its reliance solely on the protein backbone, it required them to employ phage display to optimize the combination of side chains. The strategy proposed in our study provides an alternative approach to designing phospho-specific proteins, involving the direct incorporation of protein side chains in a more rational manner.

To summarize, through the comparison of two antibodies that display high-binding affinity and selectivity with non-antibody proteins, we have revealed that antibodies recognize the phosphate group using a basic side chain and many polar, uncharged amino acids in a stereo-specific manner. This mechanism is significantly different from that of non-antibody proteins, which exhibit lower binding affinities. Our results highlight how MD simulations provide insights into the dynamic recognition of phosphorylated epitopes, revealing key atomic-level interactions that drive the high affinity and selectivity of antibodies.

## Experimental procedures

### Peptide synthesis

All the peptides used in this study were manufactured by SCRUM Inc., and correct synthesis was confirmed by MS analysis. For immunization and Fab screening, the synthesized peptides were conjugated to carrier protein KLH. Peptide sequences used in this study are as follows: phosphorylated Akt peptide, RPHFPQF(pS)YSAS and Akt peptide, RPHFPQFSYSAS.

### Library construction and selection and Fab screening

Library construction from the bone marrow and spleen cells obtained from immunized rabbits and Fab screening were conducted as described in a previous study (25). Briefly, total RNA was obtained using TRI reagent followed by cDNA synthesis. Antibody genes were amplified by PCR and incorporated into a phagemid vector (26). The library DNA was electroporated into *E. coli* XL-1 Blue cells followed by VCS M13 helper phage infection, and phage production was induced in the presence of 1 mM of isopropyl-1-thio-β-D-galactopyranoside. Phage was precipitated from the bacterial supernatant with PEG/NaCl and resuspended in 1% BSA/PBS. The Fab clones were selected by three rounds of biopanning using microtiter wells, and selected antibody sequences were analyzed. The binding of selected Fab to peptides was evaluated by ELISA.

### scFv and Fab preparation as recombinant protein

Recombinant scFv proteins were expressed and purified by the *E. coli* expression system following a previous study (27). Briefly, gene fragments encoding valuable regions of heavy and light chains from selected Fab clones were cloned into pRA2, an expression vector for the *E. coli* expression system (28). *E. coli* BL21(DE3) cells were transformed with the resultant expression vectors, and protein expression was induced by the addition of 0.5 mM isopropyl-1-thio-β-D-galactopyranoside. The cells were harvested, and the expressed proteins were extracted by sonication.

A recombinant Fab protein of antibody C7 was expressed and purified by the Expi293 expression system (Thermo Fisher Scientific) following a previous study (29) concerning the manufacturer’s standard protocol. Briefly, gene fragments encoding the heavy chain with a His_6_ tag or the light chain were cloned into pcDNA3.4 vectors (Thermo Fisher Scientific). The supernatant was harvested after 5 days of culturing.

The proteins were purified by Ni-NTA resin (QIAGEN) followed by size-exclusion chromatography using a HiLoad 16/600 Superdex 75 pg column (Cytiva). The monomer peak fractions were collected.

### Isothermal titration calorimetry

The thermodynamic parameters of interactions between peptides and scFvs were evaluated using an iTC200 microcalorimeter (Malvern Panalytical). Samples were dialyzed against PBS. In each experiment, 5 μM of a scFv was loaded into the cell and 50 μM of a peptide into the syringe. The thermodynamic parameters were calculated by the fitting of titration curves using ORIGIN 7.0 software (MicroCal), and triplicate analyses were conducted for each WT and mutant.

### Crystallization, data collection, and refinement

Purified scFvs and a Fab were mixed with the phosphorylated peptide, and the initial crystallization screening was carried out using an Oryx8 protein crystallization robot (Douglas Instruments). Single crystals for each scFv or Fab were obtained in a solution of 0.2 M lithium chloride, 20% w/v polyethylene glycol 3350, pH 8.0 (A4 scFv), 0.2 M ammonium sulfate, 30% polyethylene glycol 4000, 20 mM Tris HCl, 20 mM NaCl, pH 8.0 (C7 scFv), and 0.2 M sodium chloride, and 19% polyethylene glycol 3350, pH 8.0 (C7 Fab). Suitable crystals were harvested, briefly incubated in mother liquor supplemented with cryoprotectant, and transferred to liquid nitrogen for storage until data collection.

Diffraction data from single crystals obtained as explained above were collected in beamlines BL5A and AR-NW12 of the Photon Factory. Diffraction images were processed with the program MOSFLM and merged and scaled with the program SCALA or AIMLESS (30) of the CCP4 suite (31). The structures of A4 and C7 scFv were determined by the molecular replacement method using the coordinates of a rabbit Fab (32) (PDB entry code 4HBC) with the program PHASER (33). The structure of C7 scFv and Fab was determined by the molecular replacement method using the coordinates of scFv A4 from above and those of antibody E6 (PDB entry code 6LDX) (25). The models were refined with the programs REFMAC5 (34) and built manually with COOT (35). Validation was carried out with PROCHECK (36). Data collection and structure refinement statistics are given in Table S3.

### MD simulations

MD simulations of the antibody-phosphorylated peptide complexes were performed using GROMACS 2016.3 (37) with the CHARMM36 m force field (38). The complex structures were solvated with TIP3P water (39) in a rectangular box such that the minimum distance to the edge of the box was 15 Å under periodic boundary conditions through the CHARMM-GUI (13). The protein charge was neutralized with added Na or Cl, and additional ions were added to imitate a salt solution of concentration 0.14 M. Each system was energy-minimized for 5000 steps with the steepest descent algorithm as implemented in the GROMACS and equilibrated with position restraints of protein heavy atoms and the NVT ensemble (298 K) for 1 ns. Further simulations were performed with the NPT ensemble at 298 K for 210 ns. The time step was set to 2 fs throughout the simulations. A cutoff distance of 12 Å was used for Coulomb and van der Waals interactions. Long-range electrostatic interactions were evaluated using the particle mesh Ewald method (40). Covalent bonds involving hydrogen atoms were constrained by the LINCS algorithm (41). A snapshot was saved every 100 ps.

For each complex, we used both the PO_3_^2−^ and the PO_3_H^−^ protonation states of the phosphoserine residue. Each simulation was conducted three times, each at a different initial velocity (total simulation time, 6 μs). UCSF Chimera (42) was used to render the molecular graphics. According to a previous study (10), we defined a protein residue as interacting with the phosphoserine if the distance between the phosphorus atom and any atom of the residue was less than 4.0 Å. Trajectories of non-antibody proteins were taken from the previous study (10) for reassessment.

## Data availability

The coordinates and structure factors for the structure of antibodies in complex with phosphorylated peptide have been deposited in the PDB under entry codes 8JOW (A4 scFv/phosphorylated peptide) 8ZPU (C7 scFv/phosphorylated peptide) and 8ZXW (C7 Fab/phosphorylated peptide).

## Supporting information

This article contains supporting information.

## Conflict of interest

The authors used the patented technology Wiz-Amp (US 9,890,414), invented by S. C. J. O. and T. M. for antibody acquisition.
