# Supplementary material for: Unveiling the structural mechanisms behind high affinity and selectivity in phosphorylated epitope-specific rabbit antibodies
Source: J Biol Chem. 2024 Nov 13;300(12):107989. doi: 10.1016/j.jbc.2024.107989 (PMC11681868; doi:10.1016/j.jbc.2024.107989)
Supplement: Supplementary Figures [file mmc1.pdf]

Supplementary Figures.

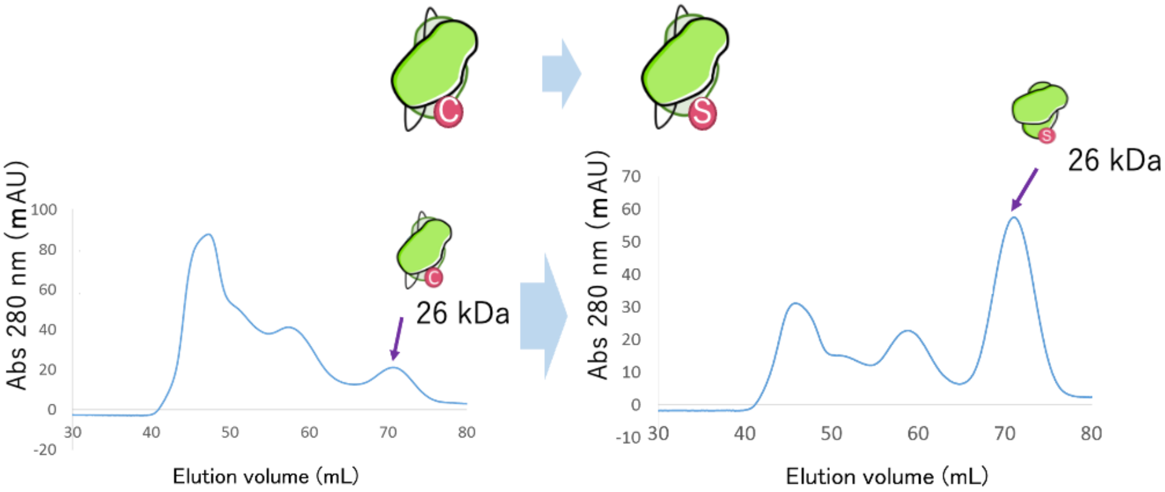

Figure S1. Elution curves of the size exclusion chromatography (SEC) of scFv (A4) with Cys80 (Left) and Cys80Ser (Right).

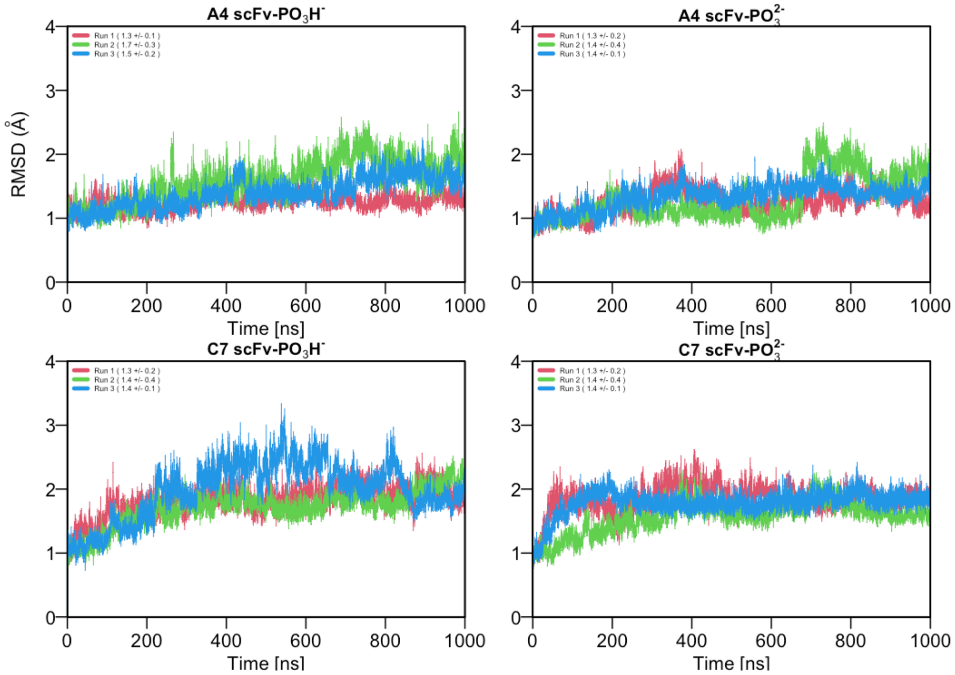

Figure S2. Cα-RMSDs of A4 scFv and C7 scFv excluding the GS linkers and the 5 N-terminal and C-terminal residues. Different colors represent different replicates of the simulations.

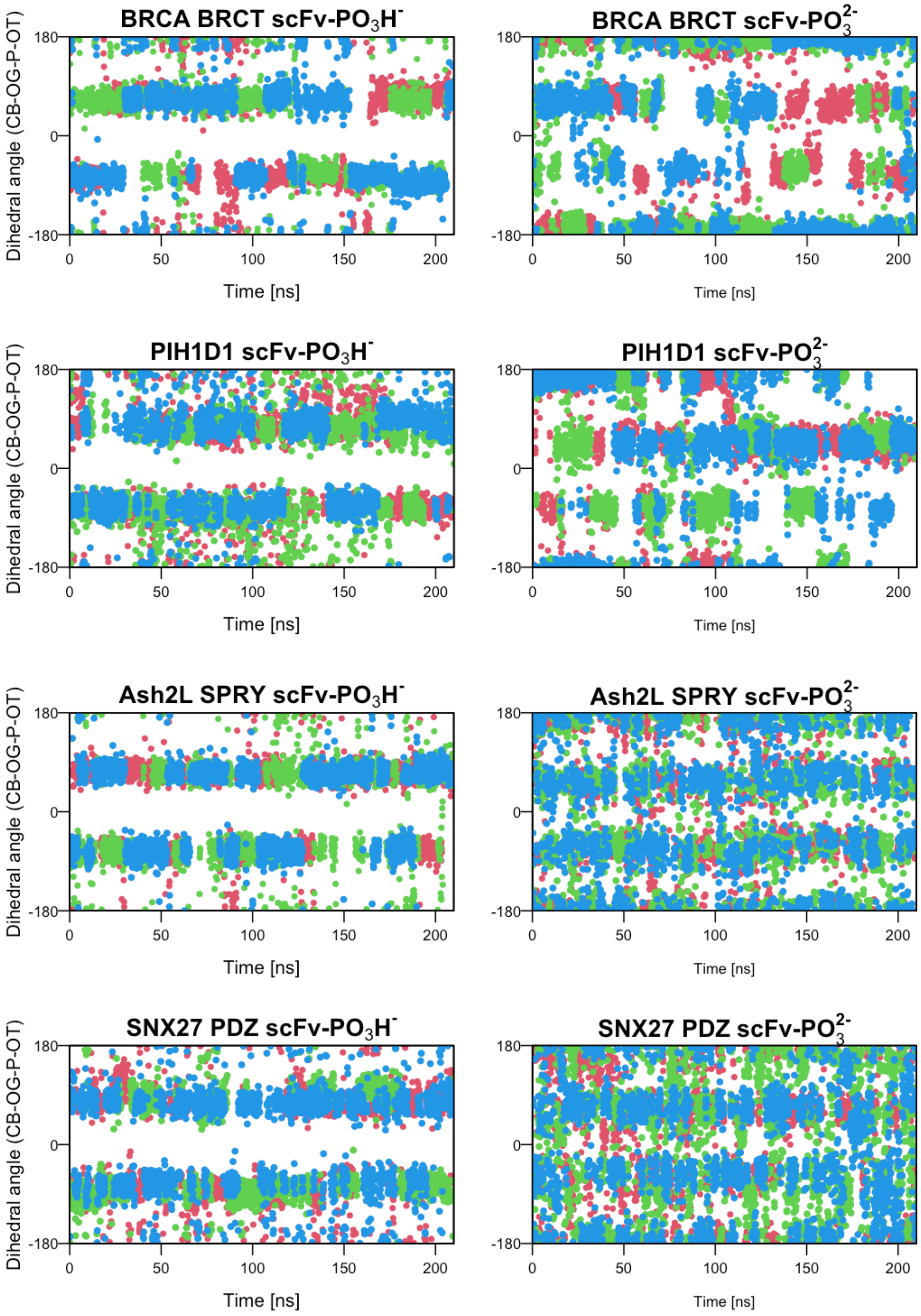

Figure S3. Time evolution of the dihedral angle consisting of CB, OG, P and OT atoms of the phosphate group during the simulations of non-antibody proteins. MD trajectories were taken from a previous study (10). Different colors represent different replicates of the simulations.
