# Supplementary material for: Unveiling the structural mechanisms behind high affinity and selectivity in phosphorylated epitope-specific rabbit antibodies
Source: J Biol Chem. 2024 Nov 13;300(12):107989. doi: 10.1016/j.jbc.2024.107989 (PMC11681868; doi:10.1016/j.jbc.2024.107989)
Supplement: Supplementary Tables [file mmc2.pdf]

Supplementary Tables.

Table S1. ELISA signals (A450) against phosphorylated antigen and non-phosphorylated antigen by 4 rabbit monoclonal antibodies obtained in this study.

Clone #	Phosphorylated-peptide	Non-phosphorylated-peptide
Ramos-C7	3.69	0.072
Ramos-G11	3.76	0.11
Ramos-A3	3.61	0.29
Ramos-A4	3.61	0.18

Table S2. Parameters of ITC about the interaction between the 4 rabbit monoclonal antibodies and phosphorylated antigen or non-phosphorylated antigen.

Antibody	Parameters	Phosphorylated-peptide	Non-phosphorylated-peptide
A3 scFv	K_D	31.8 nM	Did not bind
	ΔH	−18.5 kcal/mol	
	$T\Delta S$	−8.3 kcal/mol	
A4 scFv	K_D	3.34 nM	1.12 μM
	ΔH	−24.4 kcal/mol	−8.56 kcal/mol
	$T\Delta S$	−12.8 kcal/mol	−0.42 kcal/mol
G11 scFv	K_D	18.1 nM	Weak binding
	ΔH	−21.6 kcal/mol	
	$T\Delta S$	−11.0 kcal/mol	
C7 scFv	K_D	26.9 nM	Did not bind
	ΔH	−14.4 kcal/mol	
	$T\Delta S$	−4.0 kcal/mol	

Table S3. Data collection and refinement statistics. Statistical values given in parenthesis refer to the highest resolution bin.

	A4 scFv + peptide	C7 scFv + peptide	C7 Fab + peptide
Space Group	P 1	P 3_2 2 1	P 1
Unit cell			
a, b, c (Å)	43.5, 43.6, 63.5	126.6, 126.6, 82.4	54.4, 67.8, 71.3
α, β, γ (°)	71.3, 84.4, 87.6	90.0, 90.0, 120.0	112.5, 105.0, 98.3
Resolution (Å)	41.3 – 1.40 (1.47 – 1.40)	49.8 – 2.80 (2.85 – 2.80)	48.0 – 1.33 (1.35 – 1.33)
Wavelength	1.0000	1.0000	1.0000
Observations	144,045 (19,558)	210,688 (30,951)	656,828 (22,144)
Unique reflections	77,744 (10,677)	19,037 (2,756)	188,006 (6,654)
$R_{merge.}$	0.092 (0.368)	0.176 (1.030)	0.043 (0.463)
$R_{p.i.m.}$	0.092 (0.368)	0.055 (0.321)	0.035 (0.383)
$CC_{1/2}$	0.977 (0.650)	0.996 (0.807)	0.998 (0.781)
$I / \sigma (I)$	5.7 (2.1)	12.8 (2.6)	15.4 (2.2)
Multiplicity	1.9 (1.8)	11.1 (11.2)	3.5 (3.3)
Completeness (%)	88.8 (83.4)	99.8 (100.0)	93.5 (66.6)
**Refinement Statistics**			
Resolution (Å)	41.3 – 1.40	49.8 – 2.80	49.8 – 1.33
R_{work} / R_{free} (%)	16.2 / 19.9	30.4 / 34.5	13.7 / 16.5
No. complexes	2	2	2
No. atoms			
Antibody	3,402	3,460	6,536
Peptide	205	142	152
Other	2	32	7
Water	463	14	858
B-factor (Å^2)			
Antibody	11.9	45.3	20.6
Peptide	19.4	64.0	24.3
Other	15.2	55.4	27.0
Water	19.6	21.9	29.8
Ramachandran Plot			
Preferred (%)	90.8	86.3	92.4
Allowed (%)	7.6	13.2	7.1
Outliers (%)	1.6	0.5	0.4
RMSD Bond (Å)	0.009	0.005	0.008
RMSD Angle (°)	1.61	1.37	1.53
PDB entry code	8JOW	8ZPU	8ZXW

Table S4. Frequency of bidentate interactions between an Arg residue (H-R95 and H-R53 for A4 and C7, respectively) and the phosphate group. Frequencies of the three independent simulations (800 ns each) were averaged.

	PO_3H^- (%)	PO_3^{2-} (%)
A4 (H-R95)	8.5	34.1
C7 (H-R53)	97.4	100
